# The identity of belowground herbivores, not herbivore diversity, mediates impacts on plant productivity

**DOI:** 10.1038/srep39629

**Published:** 2016-12-22

**Authors:** Ivan Milosavljević, Aaron D. Esser, Nilsa A. Bosque-Pérez, David W. Crowder

**Affiliations:** 1 Washington State University Entomology, 166 FSHN Bldg, Pullman, WA, 99164, USA; 2 Washington State University Extension, 205 W Main, Ritzville, WA, 99169, USA; 3 Department of Plant, Soil and Entomological Sciences, University of Idaho, 875 Perimeter Drive, Moscow, ID, 83844-2339, USA.

## Abstract

Across many ecosystems, increases in species biodiversity generally results in greater resource acquisition by consumers. Few studies examining the impacts of consumer diversity on resource capture have focused on terrestrial herbivores, however, especially taxa that feed belowground. Here we conducted field mesocosm experiments to examine the effects of variation in species richness and composition within a community of wireworm herbivores on wheat plant productivity. Our experiments involved wireworm communities consisting of between one and three species, with all possible combinations of species represented. We found that the presence of wireworms reduced plant biomass and seed viability, but wireworm species richness did not impact these plant metrics. Species identity effects were strong, as two species, *Limonius californicus* and *Selatosomus pruininus*, had significantly stronger impacts on plants compared to *L. infuscatus*. Communities with either of the two most impactful species consistently had the greatest impact on wheat plants. The effects of wireworms were thus strongly dependent on the particular species present rather than the overall diversity of the wireworm community. More broadly, our study supports the general finding that the identity of particular consumer species within communities often has greater impacts on ecosystem functioning than species richness.

Across a diverse array of ecosystems, greater consumer species richness generally increases the consumption of shared resources^1^,^2^,^3^,^4^,^5^. This positive relationship between biodiversity and resource capture is often attributed to one of two mechanisms, species complementarity or species identity effects^4^,^5^,^6^,^7^. Species complementarity occurs when consumer species feed on unique resources in space or time, such that more diverse communities consume more resources by feeding across diverse niches^4^,^5^,^6^,^7^. Species identity effects or ‘sampling effects’, in contrast, arise when more species-rich communities are more likely to contain highly impactful (i.e., species that consume significantly more resources than an average species) species by chance alone, resulting in greater resource consumption^4^,^5^,^6^,^7^.

One approach to demonstrate complementarity among consumer species is to explore the niche breadth of each species and identify unique niches available to each^8^; if species occupy distinct niches, complementarity can occur^8^. Another approach is to directly manipulate niche complementarity among consumers, and experimentally determine if greater complementarity promotes resource consumption^9^,^10^. Both of these approaches, however, require complex experimental designs that are often not practical for many systems. A more common approach is to compare resource consumption in diverse consumer communities with that of the single most impactful species^6^,^7^. With this approach, if the diverse consumer community significantly depletes resource levels below that of the most impactful species, complementarity is inferred^6^,^7^, but if not then identity effects are inferred.

While empirical studies of consumer diversity and resource consumption have identified both species complementarity and identity effects^1^,^2^,^3^,^4^,^5^, meta-analyses have shown that species identity effects are more prevalent^1^. To date, however, studies on the consumer diversity-resource consumption relationship have focused almost exclusively on predator-prey or aquatic systems^1^. Few studies have focused on interactions between herbivore diversity and plant productivity in terrestrial ecosystems. Thus, whether patterns of diversity strengthening resource consumption seen in predator-prey^1^,^2^,^3^,^4^,^5^,^6^,^7^,^8^,^9^,^10^,^11^,^12^ and aquatic^13^,^14^,^15^,^16^ systems, and the underlying mechanisms, hold for terrestrial herbivore-plant systems remains largely unknown.

Here we investigated the relationship between herbivore diversity and plant productivity in a system consisting of wireworms and wheat plants. Wireworms, the larvae of click beetles (Coleoptera: Elateridae), are a group of generalist herbivores that feed voraciously belowground on the seeds, roots, and stems of wild and cultivated plants. Wireworms cause significant economic damage to crops and natural systems across a wide range of climates^17^,^18^,^19^,^20^. In wheat cropping systems of the Pacific Northwestern United States, wireworms have become the most economically damaging insect pest^18^,^19^. The distribution of wireworm species, and the composition of wireworm communities, varies considerably across regions based on environmental conditions and crop type^17^,^18^. In agricultural ecosystems, for example, many fields often contain a single wireworm species, while others in the same region contain a diverse mixture of species (although the mechanisms driving this variability remain largely unknown)^18^. However, despite this variability, the impact of variation in wireworm species diversity and identity on plants remains virtually unknown, although there is reason to expect that this variability is biologically important. For example, wireworm species can differ greatly in their feeding ecology^17^,^19^,^20^. Different species can feed at varying depths within the soil profile^18^, which might lead to spatial niche partitioning between species. Moreover, different species vary in their seasonal activity patterns^17^,^19^,^20^, which might promote temporal niche partitioning among species. Thus, we hypothesized that greater wireworm diversity might increase plant resource consumption through spatial and/or temporal niche partitioning.

We conducted a field mesocosm experiment to test whether the diversity and composition of wireworm communities mediated their impacts on wheat plant productivity. Our experiments involved the three most common species in agricultural fields of Washington State, USA; these species accounted for nearly 90% of wireworms collected in regional surveys of 160 fields throughout the Pacific Northwest^18^. These three species are responsible for inflicting significant economic damage to cereal crops in the Pacific Northwest, with up to 70% yield losses in highly infested fields^18^. We tested whether the impacts of the three wireworm species on wheat plants differed when they were present singly or in diverse communities. By using a substitutive experimental design we also isolated and measured wireworm identity and diversity effects. Our results shed light on the effects of belowground herbivore diversity and species identity on plant productivity.

## Results

### Effects of herbivore presence and diversity on wheat productivity

**Figure 1:**
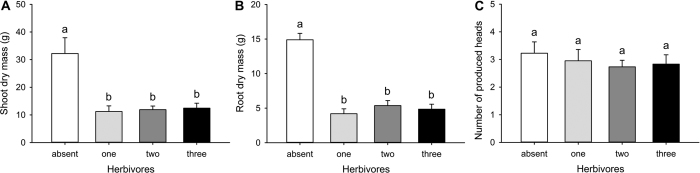
Effects of wireworm presence and species richness on wheat productivity (plant metrics). Shown are the mean (**A**) shoot (aboveground) dry mass, (**B**) root dry mass, and (**C**) the number of produced seed heads (+SE) in control treatments (wireworms absent) and treatments with one, two, or three species (values were pooled across unique compositions at each richness level). Within each panel, different letters above the bars indicate significant differences based on a posthoc Dunn’s tests (α = 0.05).

**Figure 2:**
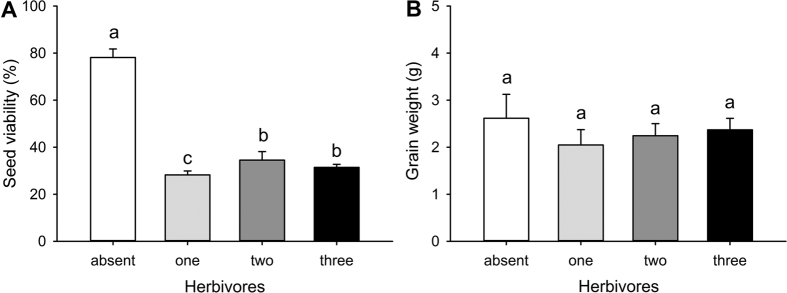
Effects of wireworm presence and species richness on wheat productivity (seed metrics). Shown are the mean (**A**) seed viability and (**B**) grain weight (+SE) in control treatments (wireworms absent) and treatments with one, two, or three species (values were pooled across unique compositions at each richness level). Within each panel, different letters above the bars indicate significant differences based on posthoc pairwise likelihood ratio contrasts for seed viability and posthoc Dunn’s tests (α = 0.05) for grain weight.

We conducted a field mesocosm experiment to determine the impacts of wireworm species richness and identity on wheat plant productivity. The presence of wireworms significantly reduced plant biomass both above- and belowground (Fig. 1A,B; Table 1). Seed viability was also significantly reduced when wireworms were present (Fig. 2A; Table 1). Other metrics of plant productivity (the number of produced seed heads, grain weight) were not significantly impacted by the presence of wireworms (Figs 1 and 2; Table 1).

While the presence of wireworms strongly impacted plant productivity, wireworm species richness did not significantly impact aboveground (Fig. 1A; Table 1) or root biomass (Fig. 1B; Table 1). Seed viability, however, was significantly reduced when wireworms were present as single species compared to within diverse communities (Fig. 2A; Table 1). The number of produced seed heads and grain weight were not significantly impacted by wireworm species richness (Figs 1 and 2; Table 1).

### Examining effects of wireworm species identity and complementarity

**Figure 3:**
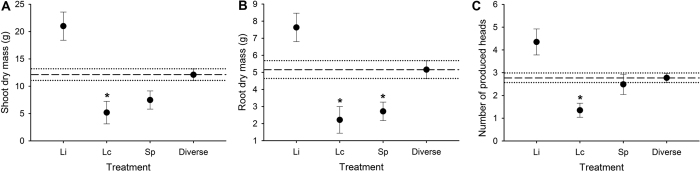
Plant productivity (plant metrics) when wireworms were present singly compared with the productivity across diverse communities (averaged over two- and three-species treatments). Shown are the mean (**A**) shoot (aboveground) dry mass, (**B**) root dry mass, and (**C**) the number of produced seed heads (±SE) for *L. infuscatus* (Li), *L. californicus* (Lc), *S. pruininus* (Sp), and diverse treatments. The long dashed lines indicate the mean productivity averaged across the diverse communities, while dotted lines represent ±1 SE from this mean. Within each panel, asterisks indicate that the single-species caused significantly more damage to wheat plants than the average of the diverse treatments (based on one sample *t*-tests, α = 0.05).

**Figure 4:**
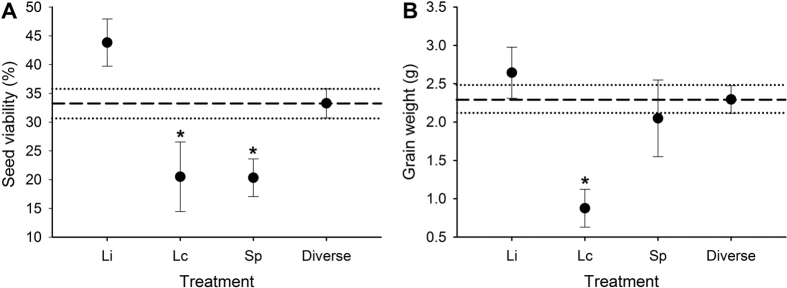
Plant productivity (seed metrics) when wireworms were present singly compared with the productivity across diverse communities (averaged over two- and three-species treatments). Shown are the mean (**A**) seed viability and (**B**) grain weight (±SE) for *L. infuscatus* (Li), *L. californicus* (Lc), *S. pruininus* (Sp), and diverse treatments. The long dashed lines indicate the mean wheat productivity averaged across the diverse communities, while dotted lines represent ±1 SE from this mean. Within each panel, asterisks indicate that the single-species caused significantly more damage to wheat plants than the average of the diverse treatments (based on one sample *t*-tests, α = 0.05).

**Figure 5:**
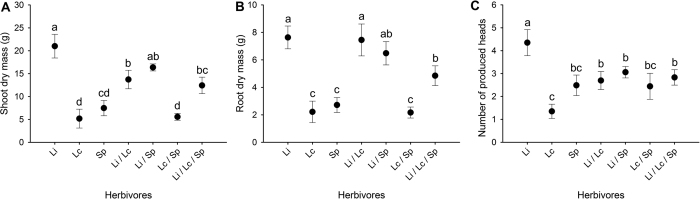
Plant productivity (plant metrics) when wireworms were present singly compared with the productivity in two- and three-species treatments. Shown are the mean (**A**) shoot (aboveground) dry mass, (**B**) root dry mass, and (**C**) the number of produced seed heads (±SE) for *L. infuscatus* (Li), *L. californicus* (Lc), *S. pruininus* (Sp), *L. infuscatus* + *L. californicus* (LiLc), *L. infuscatus* + *S. pruininus* (LiSp), *L. californicus* + *S. pruininus* (LcSp) and *L. infuscatus* + *L. californicus* + *S. pruininus* (LiLcSp) treatments. Dotted lines represent ±1 SE from means. Within each panel, different letters above the bars indicate significant differences based on posthoc Dunn’s tests (α = 0.05).

**Figure 6:**
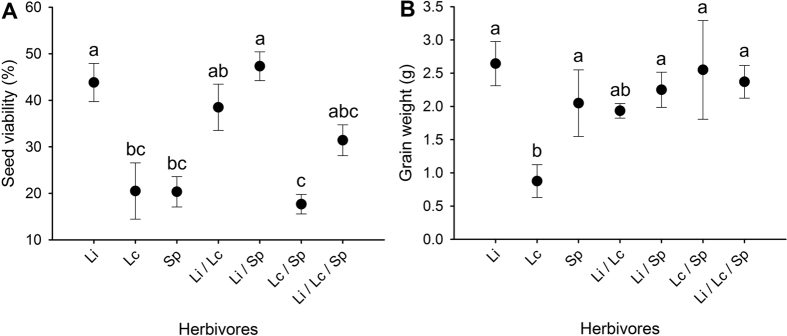
Plant productivity (seed metrics) when wireworms were present singly compared with the productivity across diverse communities (averaged over two- and three-species treatments). Shown are the mean (**A**) seed viability and (**B**) grain weight (±SE) for *L. infuscatus* (Li), *L. californicus* (Lc), *S. pruininus* (Sp), *L. infuscatus* + *L. californicus* (LiLc), *L. infuscatus* + *S. pruininus* (LiSp), *L. californicus* + *S. pruininus* (LcSp) and *L. infuscatus* + *L. californicus* + *S. pruininus* (LiLcSp) treatments. Dotted lines represent ± 1 SE from this mean. Within each panel, different letters above the bars indicate significant differences based on posthoc pairwise likelihood ratio contrasts for seed viability and posthoc Dunn’s tests (α = 0.05) for grain weight.

We compared the impacts of single wireworm species to diverse communities on wheat plants to determine if variation across our treatments was due to species identity or complementarity effects. We found no significant differences between observed plant productivity in diverse communities and the expected productivity based on the averages of each wireworm species present singly (Table 2). This provides further evidence that wireworm diversity did not impact plant productivity. However, we found that two wireworm species, *L. californicus* and *S. pruininus*, had significantly greater negative impacts on plant productivity when present alone or in a two-species community with only these two species, compared to other wireworm compositions (Table 2, Figs 3, 4, 5 and 6). We observed significant species identity effects for above- and belowground biomass, the number of produced seed heads, seed viability, and grain weight (Table 2). For each of these metrics, *L. infuscatus* was the least impactful species, while *L. californicus* and *S. pruininus* were significantly more impactful when present alone or in a two-species community without *L. infuscatus* (Figs 3, 4, 5 and 6). Two-species communities with *L. infuscatus* and either *L. californicus* or *S. pruininus* consistently had significantly lower impacts than communities with *L. californicus* and *S. pruininus* (Figs 3, 4, 5 and 6).

## Discussion

Over the past several decades a considerable amount of empirical research has focused on the effects of species diversity on ecosystem functioning^1^,^2^,^3^,^4^,^5^. Studies investigating consumer diversity and resource capture have generally demonstrated strong positive impacts of diversity, although more evidence exists for species identity effects than complementarity as the primary underlying mechanism^1^. For example, numerous multi-predator studies have found that greater predator diversity enhances the suppression of prey resources because the most voracious predators are more likely to be present in diverse compared with simple communities^1^,^4^,^21^. The biodiversity of aquatic herbivores has also been shown to generally promote consumption of plant tissue due to strong identity effects^13^,^14^,^15^,^16^. However, to date few studies have examined effects of the diversity of terrestrial consumer communities on plant consumption.

Our results show that the identity of herbivorous wireworm species mediated impacts on wheat productivity. Wireworms were impactful herbivores in our experiments, reducing plant biomass and reproductive capacity. These direct impacts on yield observed here might be further magnified in the field situations, as plants with less developed root systems are more susceptible to lodging^22^, an indirect effect of wireworm feeding. However, we did not find that wireworm diversity increased the depletion of wheat plant resources. Rather, two impactful wireworm species, *L. californicus* and *S. pruininus,* caused greater deleterious effects on wheat productivity compared to diverse communities or a third species, *L. infuscatus*. Moreover, two-species communities containing both *L. californicus* and *S. pruininus* exerted stronger deleterious effects on multiple plant productivity metrics than other diverse communities that included *L. infuscatus*. The initial abundance of *L. californicus* and *S. pruininus* was lower in the two- or three-species communities containing *L. infuscatus* due to our substitutive design, which may have in turn weakened impacts on plants.

Previous work shows that the distribution of wireworm species, and the composition of wireworm communities, differs considerably across agroecosystems of the Pacific Northwestern United States based on environmental variation^18^,^19^. Although little is known about the feeding ecology of *S. pruininus*, *L. californicus* and *L. infuscatus* differ significantly in their feeding modes^19^. *Limonius infuscatus* actively feeds on wheat plants primarily from seeding (typically April or May) through the month of June, after which it declines in activity (although some feeding occurs throughout the season), whereas *L. californicus* larvae remain highly active for the entire season, from seeding through harvest (typically August or September)^19^. We thus expected that temporal niche partitioning between different wireworm species might lead to stronger impacts on plants when wireworms were present in diverse communities. Different wireworm species also can feed at varying depths within the soil profile^19^, which could promote spatial niche partitioning when wireworms were present in diverse communities. However, we found that the biodiversity of wireworm communities did not increase depletion of wheat plant resources in our mesocosms.

It is possible that the nature of our mesocosms, which contained only a single plant species as resource, obscured potential complementarity between wireworm species. Previous work in predator-prey systems, for example, has shown that the diversity of the resource base can have considerable impacts on the biodiversity-ecosystem functioning relationship. In a system with predators feeding on aphids on collard plants, for example, coccinellid beetles forage on leaf edges while parasitoid wasps forage on leaf centers, leading to spatial complementarity and a positive relationship between predator diversity and aphid consumption^10^. However, when caterpillars are introduced to the system this complementarity is reduced because caterpillars chew holes in leaf centers (creating edges for coccinellids), and species identity effects become prevalent due to increased niche overlap between predators^10^. Similarly, Finke and Snyder^9^ demonstrated positive effects of consumer complementarity on resource consumption only when the resource base was diverse. In that study, the authors took advantage of host fidelity between parasitoid wasps and aphids, which allowed them to independently manipulate predator niche breadth (generalists vs. specialists) and resource (aphid) diversity. They found that resource exploitation increased only with greater diversity of specialist parasitoids when different species consumed different aphid species, demonstrating strong complementary effects^9^. Conversely, our study manifested a high degree of niche overlap due to the presence of a single plant resource, potentially diminishing the ability of wireworms to partition resources. However, further research should be carried out in order to verify such hypothesis.

Our results support large-scale field trials showing that *L. californicus* is a more damaging species for commercial wheat producers in the Pacific Northwestern United States than *L. infuscatus*^23^. *Limonius californicus* feeds actively throughout the season, while *L. infuscatus* is limited in activity to early months, which could contribute to the greater damage potential of *L. californicus* over the course of an entire growing season. *L. californicus* is also less impacted by seed-applied insecticides than *L. infuscatus*^19^,^23^, which can lead to greater damage caused by *L. californicus.* It remains unclear, however, whether *S. pruininus* causes more or less damage to commercial wheat crops than the two *Limonius* species. This is because the *Limonius spp.* were believed to be the primary economically important species in the Pacific Northwest^23^, and thus research to date focused primarily on these species. Only recently, when large-scale surveys were performed throughout this region, was *S. pruininus* discovered in high abundance^18^; however, detailed studies of the economic significance of this pest have yet to be performed and were one motivating reason for this study. However, the spatial distribution of wireworm species tends to remain fairly consistent within fields over multiple seasons^18^. Sampling can thus provide producers with a reliable indicator of which species to expect in order to guide management.

While our results suggest that *L. californicus* and *S. pruininus* likely require more aggressive management than *L. infuscatus*, our experiments were conducted at a single level of wireworm abundance. Variation in wireworm abundance, in conjunction with the species present, would likely ultimately dictate the most successful management strategy. Indeed, studies of consumer diversity and resource consumption have shown that abundance may mediate the impacts of biodiversity^8^. When abundance of consumers is low, competition is relaxed and the impacts of diversity might be marginalized because there may be enough resources available even for species competing in the same niche. However, when consumer density is high, resource competition intensifies and only species that occupy distinct niches will contribute additively to resource consumption (i.e., a positive effect of biodiversity can occur)^8^. It is possible that at higher wireworm densities we would have observed stronger diversity effects than were observed here. Future work should attempt to determine how field-level variation in wireworm abundance, diversity, and species composition mediate the productivity of wheat and other commercial crops.

Our results are in line with the broader literature on relationships between consumer biodiversity and resource acquisition which shows that the identity of herbivores is critical for plant performance and abundance^1^. Herbivorous wireworms likely pose greater threats to wheat production when communities consist of a single dominant consumer species (*L. californicus* or *S. pruininus*) or the right mixture of species (e.g., *L. californicus* and *S. pruininus*) rather than a complex of multiple species or a less impactful species (*L. infuscatus*). More studies of terrestrial herbivores would be beneficial to place our research in a broader context, but such studies are still under-represented in the literature^1^. Further examining the relationship between herbivore diversity and plant productivity in natural and managed ecosystems would provide considerable benefits for our understanding of the functioning of terrestrial ecosystems and for the development of integrated pest management strategies.

## Methods

### Experimental setup

We conducted a field mesocosm experiment to evaluate the impacts of three wireworm species, *L. infuscatus*, *L. californicus*, and *Selatosomus pruininus* on wheat plants, *Triticum aestivum*. Wheat is a predominant crop grown in the Pacific Northwestern United States and a highly suitable host for wireworms^18^,^19^,^23^. The experiment was conducted at the Washington State University Tukey Orchard in Pullman, Washington, USA. Experimental units were plastic containers (60 cm long, 45 cm wide, 40 cm deep) with small drainage holes (0.3 mm in diameter) drilled in the bottom to allow water drainage. These holes are small enough that they prevented the escape of wireworms. Cages were installed in the ground, leveling the top of the container with the soil surface, and filled with previously excavated soil.

Wheat seeds (variety Louise) used in the experiments were commercially purchased. All plants were sown in growth chambers for 7d (Feekes’ growth stage 1) with a 16:8 h light:dark period at a light intensity of 600 μE m^−2^ s^−1^ and a temperature regime of 20:18 °C (light:dark)^24^,^25^. They were then transplanted to the field mesocosms on May 7^th^, 2014 and allowed 3d to acclimate before wireworms were added. Each container received 10 wheat plants (2 rows of 5 plants) with in-row and between-row spacing of 8 and 20 cm, respectively. Plants were watered every 2 to 3d throughout the experiment.

After wheat plants had acclimated, we added wireworms to cages with the the following treatments that varied in species richness and composition: (1) control (six replicates without wireworms), (2) one wireworm species (six replicates of each of the three species present alone), (3) two wireworm species (six replicates of each of the three unique species pairs), and (4) three wireworm species (twelve replicates with all three wireworm species). We thus fully replicated all possible combinations of species composition and diversity from our community of three species. Wireworms used in our experiments were field collected within 2 wk of the initiation of the experiments using bait traps^18^ and identified to species^26^,^27^. Larvae were collected from fallowed field sat Washington State University Wilke Research and Extension Farm, Davenport, WA and Washington State University Lind Dryland Research Station, Lind, WA. All wireworms were housed without food for 10d before introducing them to field mesocosms. Twelve randomly chosen large larvae (> = 9 mm in size) were assigned to each experimental mesocosms; this density is within the normal range observed in fields reporting yield loss from wireworms^19^. Single-species treatments received 12 individuals of a single species, two-species treatments received 6 individuals of each of the two wireworm species, and three-species treatments included 4 individuals of each of the three wireworm species. This substitutive design allowed us to vary species composition and diversity with the same overall abundance in each mesocosm. Each experimental unit was maintained until the wheat was mature and ready to harvest.

### Data collection

Plants were harvested on September 27^th^ 2014 and the following five measures of plant productivity were measured: (1) aboveground dry weight (shoot dry weight), (2) belowground dry weight (root dry weight), (3) the number of produced seed heads, (4) seed viability, and (5) grain weight. All plants were first harvested at the base near the soil. The aboveground matter was collected and the number of produced seed heads was counted. The samples were then dried in an oven at 100 °C for 48 h and weighed. To calculate grain weight, all seeds from each plant were weighed^28^. Ten randomly selected seeds were then germinated on blotter paper, moistened with tap water, and placed in a growth chamber at 20 °C for 7d, after which seed viability (% germination) was assessed in accordance with procedures developed by the Association of Official Seed Analysts (AOSA)^29^. Only viable seedlings were counted^30^. To record root-biomass, we extracted root cores from the soil by sifting all the soil out of each mesocosm and washing the residual soil off with water. All roots were then air-dried for 2 h and sorted between paper towels for accurate biomass determination. Prepared samples were oven-dried at 100 °C for 48 h and then weighed.

### Data analyses

We first analyzed the effect of wireworm presence on plant productivity using non-parametric Wilcoxon rank-sum tests due to non-normality in the response variables. In these analyses wireworm presence (present or absent) was our explanatory variable and each metric of plant productivity (except seed viability) was analyzed separately as a response. This allowed us to determine whether wireworms impacted plant productivity across all levels of composition and diversity. We then used non-parametric Kruskal-Wallis tests^31^, followed by posthoc Dunn’s tests, to assess whether wireworm richness (1, 2, or 3 species) impacted each plant productivity metric except seed viability. To analyze impacts of wireworm presence and biodiversity on seed viability we used logistic regression models, where seed germination (yes or no) was the binary response. For all of these analyses, when the overall model was significant, comparisons of treatment means were performed using posthoc pairwise likelihood ratio contrasts. These analyses were conducted in JMP^32^.

To assess whether species complementarity or species identity effects were predominant, for each unique diverse assemblage *j* we measured non-transgressive (*D*_*T*_) and transgressive (*D*_*max*_) over-yielding as follows^7^:


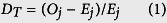



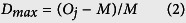


where *O*_*j*_ is the observed plant productivity in the diverse community *j*, *E*_*j*_ is the expected plant productivity of the diverse community *j* (the average productivity across all species in the community), and *M* is the plant productivity in the monoculture that had the most impactful species (in our case the wireworm species that caused the most damage to wheat). Separate analyses were conducted for each metric of plant productivity.

Non-transgressive overyielding indicates that plant productivity observed in a diverse wireworm community differed from the average productivity when these species were present singly; *D*_*T*_ thus indicates the net impact of diversity^7^. If *D*_*T*_ values are significantly below 0, it indicates that diverse wireworm communities reduced plant productivity more than species present alone; *D*_*T*_ values greater than 0 would indicate that diverse communities had significantly lower impact on plant productivity than single wireworm species. Transgressive overyielding, *D*_*max*_, occurs when the effects of a diverse community exceeds that of the most impactful species. In this case, if *D*_*max*_ values are significantly below 0, it indicates that diverse wireworm communities reduced plant productivity more than the single most impactful species present alone (i.e., transgressive overyielding occurs); *D*_*max*_ values significantly greater than 0 would indicate that the single most impactful species had greater impacts on plant productivity than a diverse community (i.e., species identity effects predominate). If both *D*_*T*_ are *D*_*max*_ are significantly negative, it would indicate a positive effect of diversity driven by species complementarity. If *D*_*T*_ is significantly negative but *D*_*max*_ is insignificant, it would indicate a positive effect of diversity driven by a species identity effect. In contrast, if *D*_*max*_ is significantly positive but *D*_*T*_ is not significant, it would indicate that species identity effects mediated impacts of wireworms on plant productivity (such that the single most impactful species exerted stronger detrimental effects on plant community than the diverse community) but that diversity itself was not significant.

We calculated *D*_*T*_ and *D*_*max*_ for each plant productivity metric (including seed viability which was treated as a percentage). We then used one-sample *t*-tests to assess whether the distribution of these values calculated across all of the unique two- and three-species communities differed significantly from 0. This analysis allowed us to detect if overyielding occurred, and whether species identity played a dominant role in wireworm communities. These analyses were conducted in JMP^32^.

## Additional Information

**How to cite this article**: Milosavljević, I. *et al*. The identity of belowground herbivores, not herbivore diversity, mediates impacts on plant productivity. *Sci. Rep.* **6**, 39629; doi: 10.1038/srep39629 (2016).

**Publisher's note:** Springer Nature remains neutral with regard to jurisdictional claims in published maps and institutional affiliations.

## Figures and Tables

**Table 1:** Effects of wireworm presence and richness on plant productivity.

Plant metric	Wireworm presence		Species richness	
	*χ*^2^	*P*	*χ*^2^	*P*
Shoot Dry Mass	13.81	0.0002	0.68	0.71
Root Dry Mass	15.72	<0.0001	1.67	0.43
Number of Produced Heads	0.51	0.47	0.40	0.82
Seed Viability	9.42	0.0021	9.22	0.01
Grain Weight	0.78	0.38	1.05	0.59

Results shown for all plant metrics except seed viability were from Wilcoxon rank-sum tests (testing effects of wireworm presence) or Kruskal-Wallis tests (testing effects of wireworm species richness); results shown for seed viability were from logistic regression models.

**Table 2:** Results of non-transgressive (*D*_*T*_) and transgressive (*D*_*max*_) overyielding analyses (one-sample *t*-tests) for multiple metrics of wheat productivity.

Plant metric	*D*_*T*_		*D*_*max*_	
	*t*	*P*	*t*	*P*
Shoot Dry Mass	0.77	0.44	6.06	<0.0001
Root Dry Mass	1.95	0.06	5.47	<0.0001
Number of Produced Heads	−0.71	0.48	3.38	0.0021
Seed Viability	1.36	0.18	3.68	0.001
Grain Weight	1.34	0.19	3.87	0.0006
